# Green synthesis of magnetic bio-Graphene nanohybrid for the immobilization of hydrolytic enzymes towards sustainable bioconversion of cellulose

**DOI:** 10.1039/d5ra06271c

**Published:** 2025-10-06

**Authors:** Christina Alatzoglou, Michaela Patila, Panagiotis G. Ziogas, Anastasia Skonta, Despoina Politi, Konstantinos Spyrou, Angela S. Kaloudi, Alexios P. Douvalis, Dimitrios P. Gournis, Haralambos Stamatis

**Affiliations:** a Laboratory of Biotechnology, Department of Biological Applications and Technologies, University of Ioannina Ioannina Greece hstamati@uoi.gr; b Physics Department, University of Ioannina Ioannina 45110 Greece; c Department of Materials Science & Engineering, University of Ioannina Ioannina 45110 Greece; d School of Chemical and Environmental Engineering, Technical University of Crete Chania 73100 Greece; e Institute of GeoEnergy, Foundation for Research and Technology-Hellas Chania 73100 Greece

## Abstract

In this work, we report a green and sustainable synthetic route for producing magnetic few-layer bio-Graphene (MbG) for the first time. Bio-Graphene (bG) was prepared *via* a green method using an aqueous olive leaf extract (OLE) as both the exfoliating and stabilizing agent, aiming to reduce the environmental impact of the traditional chemical methods. In the following step, iron oxide nanoparticles were created *in situ* on bG-OLE *via* co-precipitation using ferrous precursors. MbG was subsequently used to support the co-immobilization of cellulase (cel) and β-glucosidase (bgl), enabling the design of a recyclable, magnetically separable nanobiocatalyst. Various spectroscopic and microscopic techniques were employed to characterize the produced MbG and the resulting nanobiocatalysts. Both simultaneous and sequential immobilization strategies were applied to evaluate the synergy between cel and bgl. Several parameters were studied, such as the support-to-enzyme mass ratio, immobilization incubation time, and the order in which the enzymes were added. Although the 1-hour simultaneous co-immobilization resulted in low cel and bgl immobilization yields, the highest specific activity was observed (∼0.33 units mg^−1^). Moreover, the bi-enzymatic nanobiocatalyst demonstrated better reusability for carboxymethyl (CMC) and microcrystalline cellulose (Avicel) hydrolysis compared to the mono-enzymatic nanobiocatalyst. Subsequently, the mono- and bi-enzymatic systems were employed in continuous-flow microreactors for the hydrolysis of CMC towards glucose production. The bi-enzymatic system exhibited significantly higher turnover frequency (TOF) (0.105 h^−1^) and operational stability than the mono-enzymatic system (0.029 h^−1^). The entire synthetic route is characterized by a minimal environmental footprint, offering a platform for sustainable bioprocessing.

## Abstract 2

Magnetic bio-Graphene nanohybrid was synthesized *via* a green route using olive leaf extract and applied for the co-immobilization of cellulase and β-glucosidase, enabling continuous-flow cellulose hydrolysis.

## Graphical abstract

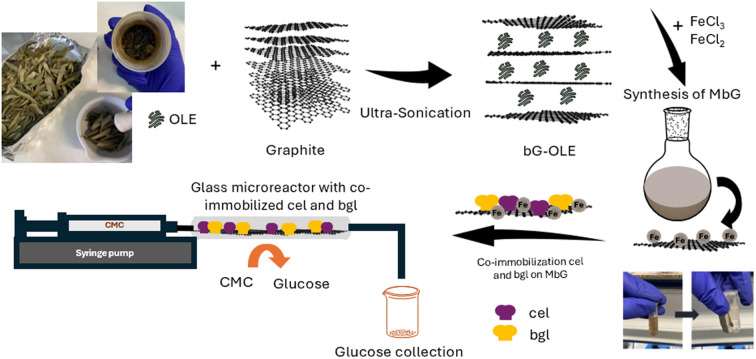

## 1. Introduction

Green-synthesized materials are preferred in several applications due to their unique physical attributes, excellent biocompatibility, and ease of fabrication.^1,2^ Bio-inspired approaches utilize non-toxic solvents, plant extracts from biomass, microorganisms, or other by-products as stabilizing and reducing agents for synthesizing materials, unlike standard procedures, which often involve hazardous chemicals, high energy consumption, and high fabrication cost.^3–6^ By avoiding the use of intensive processes and following green synthesis, which aligns with the principles of green chemistry, the environmental footprint of nanomaterial production is reduced.^7^

Plant biomass, such as olive leaves, is enriched with natural antioxidants, including polyphenols, ideal candidates for synthesizing green nanomaterials. Phytochemical-assisted liquid exfoliation of graphite in environmentally friendly solvents, such as water, can pave the way for synthesizing few-layered graphene.^8–10^ Graphite can be easily dispersed through mechanical force, such as ultrasonication, by agitating the π–π stacking interactions between graphene sheets. The aromatic rings of the phytochemicals can also boost the exfoliation process and improve the stability of the graphene dispersion through π–π stacking interactions, following eco-friendly graphene production.^11^ On the other hand, one of the standard routes for the synthesis of graphene follows the chemical reduction of graphene oxide (GO), which commonly uses toxic reducing agents like hydrazine and sodium borohydride, which are harmful to both human health and the environment due to their toxicity and the generation of hazardous waste.^12^ Phytochemicals can serve as reducing and/or capping agents for the green synthesis of magnetic nanoparticles (MNPs). They are considered non-toxic, biocompatible, and highly stable. Secondary metabolites containing alcoholic functional groups assist the bio-reduction of metal ions and play a significant role in nanoparticle formation.^13^ The magnetic properties of MNPs render them ideal candidates to prepare magnetic nanohybrids.

Attaching MNPs to carbon allotropes, such as graphene, by *in situ* co-precipitation is a promising approach for several applications.^14^ The resulting materials exhibit excellent characteristics, including a high surface area-to-volume ratio, strong mechanical performance, and superparamagnetism.^15^ The combination of carbon nanomaterials and green-synthesized MNPs has garnered attention due to their potential applications in various fields. However, only a few studies report both components being derived from green synthesis methods^16,17^ compared to chemically synthesized counterparts.^18–20^ These approaches can form more biocompatible nanosupports that can be ideal candidates for the immobilization of enzymes, enhancing their catalytic activity by creating non-denaturing interactions between the materials and the biocatalysts. Lastly, nanobiocatalytic systems based on magnetic materials allow for easy separation from reaction mixtures upon the application of an external magnetic field and can be used in various applications, including microfluidics.^21–23^

Magnetic microreactors have been widely used in the field of biocatalysis. Due to the lack of strong shear forces and relatively constant pressure, the reactor system is an ideal host for nanobiocatalysts. Applying an external magnetic field to control the behavior and the arrangement of the immobilized enzyme inside the reactor can be easily achieved, enhancing its properties.^23,24^ First, the dominance of laminar flow and diffusion-based mixing in microsystems, two major limitations of batch-scale magnetic reactors, are resolved. Secondly, the optimal system configuration can result in high conversion yields in enzymatic reactions by exploiting magnetic forces in the best possible way.^25,26^

By combining enzyme immobilization and microfluidic technology for the enzymatic hydrolysis of biomass, it is possible to avoid toxic reagents and byproducts while achieving higher conversion efficiency compared to a batch system.^4,27^ Cellulase, the key enzyme responsible for biomass degradation, consists of *endo*-glucanase, cellobiohydrolase, and β-glucosidase, and hydrolyzes the β-bond of soluble oligosaccharides, leading to the formation of glucose.^28^ However, β-glucosidase activity is phenomenally reduced compared to the other two enzymes, leading to cellobiose accumulation, inhibiting endoglucanase.^29^ Co-immobilization of exogenous β-glucosidase and cellulase can increase the glucose yield, and applying it in a high-performance reactor could further enhance the enzymatic hydrolysis process, making it an attractive route for scalable, low-waste bioprocessing.^30^

Herein, magnetic bio-Graphene (MbG) was synthesized *via* co-precipitation, using the phytochemicals attached to bio-Graphene from olive leaves as the reducing agent. This method presents a green alternative to traditional chemical synthesis routes, aiming to minimize environmental impact in accordance with the principles of green and sustainable chemistry. Various analytical, microscopic, and spectroscopic techniques characterized the resulting MbG. Next, cellulase and β-glucosidase were co-immobilized on the nanohybrid surface by physical adsorption to obtain a bi-enzymatic system with synergistic activity. Several critical parameters were investigated for preparing the magnetic bi-enzymatic nanobiocatalyst, including the MbG-to-enzymes mass ratio, the immobilization incubation time, and the sequence of enzyme addition. The optimized bi-enzymatic nanobiocatalyst was further characterized both biochemically and morphologically. Finally, immobilized cellulase and the bi-enzymatic nanobiocatalyst were employed for the first time in continuous flow microreactors to catalyze the hydrolysis of carboxymethyl cellulose, investigating the synergistic effect of both enzymes in the biocatalytic process. The entire synthetic and application approach is characterized by a minimal environmental footprint, offering a platform for sustainable and low-cost bioprocessing.

## 2. Materials and methods

### 2.1. Materials

Cellulase from *Trichoderma reesei* (consisting of endoglucanase, *exo*-cellobiohydrolase, and β-glucosidase in the ratio of 80 : 20 : 1, cel) (≥700 units g^−1^), graphite powder (<20 μm, synthetic), d(+)-glucose, carboxymethyl cellulose sodium salt (low viscosity, CMC), 3,5-dinitrosalicylic acid (DNSA), *p*-nitrophenyl-β-d-glucopyranoside (*p*NPG), bovine serum albumin (98% Fraction V, BSA), 3,4-dihydroxycinnamic acid (caffeic acid) and microcrystalline cellulose (Avicel PH101) were purchased from Sigma-Aldrich (St. Louis, MO, USA). Iron(ii) chloride hexahydrate (FeCl_2_·6H_2_O) and iron(iii) chloride tetrahydrate (FeCl_3_·4H_2_O) were obtained from ThermoFisher Scientific, and β-glucosidase from *Thermotoga maritima* (50 units mg^−1^, bgl) was procured from Megazyme (Chicago, IL, USA) and used without further purification. Sodium carbonate (Na_2_CO_3_) was purchased from Honeywell Riedel-de Haën (Seelze, Germany). All the other chemicals and reagents were of analytical grade and acquired from reliable sources. Double-distilled water (ddH_2_O) was used to prepare all the buffers and solutions.

### 2.2. Preparation of olive leaf extract

The olive leaf extract (OLE) was prepared according to previous work.^31^ Briefly, fresh *Olea europaea* leaves were collected from Serres (region of Macedonia), Greece, and properly cleaned with deionized water and shade-dried at room temperature (RT). The clean leaves were ground into a powder with a blender. Then, grounded leaves in ddH_2_O (100 g L^−1^) were heated at 90 °C and stirred for 20 min in the dark. After that period, the aqueous extract was cooled, filtered through Whatman filter paper, and centrifuged three times at 97 000*g* for 10 min. The supernatant was stored at 4 °C under nitrogen. Phytochemical analysis and determination of OLE's total phenolic content, total reducing sugars, and total protein content were carried out according to standard assays described in the SI.

### 2.3. Synthesis of bio-Graphene

Bio-Graphene (bG) was prepared according to our previous work^10^ with some modifications. Herein, OLE was used as the exfoliating and stabilizing factor. Briefly, 100 mg of graphite powder were added to 19.75 mL of ddH_2_O (final graphite concentration 5 mg mL^−1^), and 0.25 mL of OLE (final OLE concentration 0.35 mg mL^−1^) was added to the sample. The suspension was ultrasonicated for 1 h (200 watt, 20 kHz, pulser 50%) in an ice bath throughout the procedure to prevent overheating. Then, the unexfoliated graphite was precipitated by centrifugation at 335 g for 5 min, and the supernatant was separated carefully to obtain the graphitic flakes with the OLE excess (bG-OLE). Further centrifugations were performed to measure the final OLE concentration incorporated into the graphitic flakes (SI, eqn (S1)).

### 2.4. Synthesis of magnetic bio-Graphene nanohybrid

A simple co-precipitation approach was employed to synthesize MbG nanohybrid. After optimization (the optimized parameters are described in the SI), 135 mg of FeCl_2_·6H_2_O and 365 mg of FeCl_3_·4H_2_O (1 : 3 mass ratio) were dissolved in 10 mL of ddH_2_O and stirred for 15 min. Next, 15 mL of bG-OLE was added to the mixture dropwise and stirred for 30 min at 90 °C in the dark. Then, 2 M NaOH was added dropwise, and the pH was adjusted to 11.0. At this point, a change in the color of the mixture from gray to black was observed, and a black precipitate began to form. The resulting MbG was centrifuged at 121 000*g* for 15 min and rinsed five times with deionized water. The final product was left to dry at 37 °C.

### 2.5. Characterization techniques

The Ultraviolet-Visible (UV–Vis) absorption spectra of MbG samples (0.3 mg mL^−1^) were recorded on a UV–Vis spectrophotometer (Shimadzu, Tokyo, Japan) in the 200–800 nm wavelength range. The samples were dispersed in ddH_2_O, and the spectra were recorded at RT. The structural properties of the synthesized bG-OLE and MbG samples were characterized by powder X-ray diffraction (XRD), using Cu Kα radiation on a Bruker Advance D8 diffractometer. Atomic force microscopy (AFM) images were obtained in tapping mode with a 3D Multimode Nanoscope, using Tap-300 G silicon cantilevers with a tip radius of <10 nm and a force constant of ≅20–75 N m^−1^. Exfoliated nanosheets or nanoplatelets were deposited onto silicon wafers (P/Bor, single-side polished, purchased from Si-Mat) from aqueous dispersions by drop casting. The ^57^Fe Mössbauer spectrum of the MbG nanohybrid was collected in transmission geometry at RT (300 K) using a constant-acceleration spectrometer, equipped with a ^57^Co (Rh) source kept at RT. Metallic α-Fe at RT was used for the spectrometer's velocity calibration, and all isomer shift (IS) values are given relative to this standard. The spectrum was fitted and analyzed using the IMSG code.^32^ The investigation of the prepared bG-OLE and MbG samples' magnetic properties was performed on a conventional Vibrating Sample Magnetometer (VSM LakeShore 7300), through magnetization (*M*) *vs.* applied field (*H*) measurements collected at RT in fields up to 20 kOe. Fourier-transform infrared (FTIR) spectra were recorded to investigate the successful conjugation of the green synthesized MNPs on bG-OLE and to confirm cel and bgl immobilization on MbG using an FTIR-8400 infrared spectrometer (Jasco, Tokyo, Japan) equipped with a deuterated triglycine sulfate (DTGS) detector. All spectra were recorded within the 400–4000 cm^−1^ range, at 4 cm^−1^ resolution, and an average of 32 scans was applied. Samples were prepared as KBr pellets containing ∼1 wt% of the sample. X-ray photoelectron spectroscopy (XPS) measurements of MbG and the bi-enzymatic nanobiocatalyst were performed in an ultrahigh vacuum at a base pressure of 5 × 10^−9^ mbar with a SPECS GmbH spectrometer equipped with a monochromatic MgKα source (*hv* = 1253.6 eV) and a Phoibos-100 hemispherical analyzer (Berlin, Germany). The spectra were collected in normal emission, and energy resolution was set to 1.2 eV to minimize measuring time. Spectral analysis included Shirley background subtraction and peak deconvolution employing mixed Gaussian–Lorentian functions, in a least squares curve-fitting program (WinSpec) developed at the Laboratoire Interdisciplinaire de Spectroscopie Electronique, University of Namur, Belgium.

### 2.6. Optimization of mono-enzymatic nanobiocatalysts

Cel and bgl were individually immobilized onto MbG *via* physical adsorption to optimize certain parameters (MbG-to-enzyme mass ratios and immobilization incubation time), before their co-immobilization on MbG. Briefly, 3 mg of MbG was dispersed in acetate buffer (100 mM, pH 5.0) and sonicated for a short period. Firstly, the mass ratio of MbG to cel varied from 1 : 1 to 1 : 8, while MbG to bgl varied from 1 : 0.021 to 1 : 0.050. Secondly, the optimal immobilization incubation time for both enzymes was investigated (1, 2, 4, and 6 hours). All immobilization procedures were performed at 30 °C under stirring. After that, the immobilized enzymes were collected using an external magnetic field, washed three times with acetate buffer (100 mM, pH 5.0), and dried in a SpeedVac (ThermoFisher Scientific, Waltham, Massachusetts, USA). Finally, the immobilization yield was determined according to eqn (1).

Immobilization yield (%) = [(*A*_i_ − *A*_s_)/*A*_i_] × 100

where *A*_i_ and *A*_s_ are the activities (units) of the enzyme initially used for immobilization and the free enzyme collected in the supernatant, respectively. The individually immobilized enzymes will be referred to as bgl@MbG and cel@MbG.

### 2.7. Preparation of the bi-enzymatic nanobiocatalyst

The preparation conditions of the co-immobilized enzymes were based on their optimum individual immobilization conditions. In brief, the mass ratio 1 : 8 of MbG to cel and the 1 : 0.032 MbG to bgl were used for the co-immobilization. However, each enzyme in individual immobilization presented its activity peak in different incubation times (1 h for bgl and 4 h for cel). In this way, to test the ideal order that the enzymes added during the immobilization onto MbG, two strategies were followed: simultaneous co-immobilization, where both biocatalysts are immobilized onto the support material at the same time (1 h and 4 h), and sequential co-immobilization, where one biocatalyst is immobilized first, before introducing the second one (cel priority and bgl priority). Finally, the immobilization yield of enzymes in co-immobilized form was determined according to eqn (1).

### 2.8. Activity assays

The enzymatic activity of free and individually immobilized cel was assayed according to the DNSA method^33^ by measuring the amount of reducing sugars produced from the hydrolysis of CMC. Briefly, the reaction mixture consisted of 0.5 mg of cel@MbG, or an appropriate amount of soluble cel, and 0.5 mL of 1% w/v CMC solution (diluted in 100 mM acetate buffer, pH 5.0). The reaction mixture was incubated at 60 °C for 15 min. Next, MbG-Cel was separated by an external magnetic field, and 0.25 mL of the reaction mixture was mixed with 0.25 mL DNSA, followed by heating at 100 °C for 5 min. After that, 2 mL of distilled water was added to the sample. Finally, the absorbance of the samples was measured at 540 nm using a UV/Vis spectrophotometer (Shimadzu, Tokyo, Japan). One unit (U) of enzyme activity is defined as the amount of enzyme required to release one μmole of glucose from CMC.

The determination of free and individually immobilized bgl@MbG enzymatic activity was based on *p*NPG hydrolysis. For the activity assay, 0.12 or 500 μg mL^−1^ of free or bgl@MbG, respectively, was mixed with 2.0 mM *p*NPG in 100 mM acetate buffer (100 mM, pH 5.0). The reaction mixture was incubated at 50 °C for 5 min. After that, 1% w/v Na_2_CO_3_ was added to measure the released *p*-nitrophenol (*p*NP) at 410 nm using a UV/Vis spectrophotometer (Shimadzu, Tokyo, Japan). The enzyme activity was calculated *via* a pNP standard curve. One unit (U) of enzyme activity is defined as the amount of enzyme required to release one μmole of *p*NP from *p*NPG.

In the case of the bi-enzymatic nanobiocatalyst (cel/bgl@MbG), the activity was monitored by using CMC as the initial substrate, as mentioned before (cel activity assay).

### 2.9. Effect of temperature and pH of free and immobilized forms

CMC and *p*NPG enzymatic hydrolysis occurred at different temperatures (40–70 °C) for free and immobilized forms (individually immobilized and co-immobilized enzymes) in an acetate buffer (100 mM, pH 5.0). Enzyme activity was measured as previously described. The activity was determined and expressed as a percentage of relative activity according to the following eqn (2)2
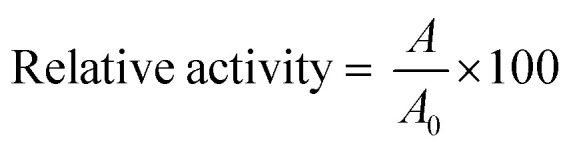
where *A* represents the activities of the enzyme at any pH or temperature, and *A*_0_ is the highest enzyme activity at all pH or temperature values, respectively.

### 2.10. Reusability of the prepared nanobiocatalysts

To evaluate the recycling of enzymes in immobilized and co-immobilized form, the nanobiocatalysts (1 mg mL^−1^) were subjected to a hydrolysis reaction of CMC and Avicel 1% w/v. After each cycle (15 min for CMC and 24 h for Avicel), the magnetic nanobiocatalyst was separated using an external magnet, washed thrice with acetate buffer, and re-suspended in a fresh substrate solution to initiate a new catalytic cycle. The total reducing sugar amount was determined using the DNSA method. The residual activity of each enzyme (%) was defined as the ratio of the remaining activity at each cycle to the activity of the first cycle.

### 2.11. Set up, optimization, and operational stability of the developed enzymatic microreactors

**Fig. 1:**
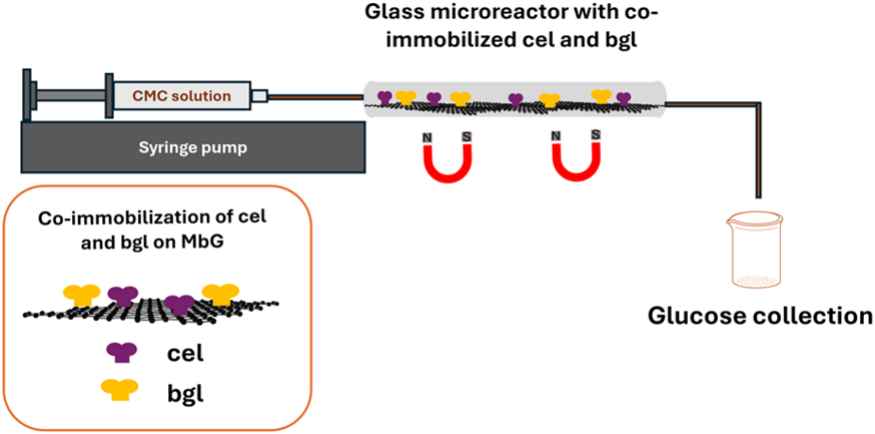
Setup of the continuous flow microreactor. A magnet stabilized the nanobiocatalyst in the microreactor.

Glass capillary microreactors (3 cm in length, 1 mm in diameter, 20 μL capacity) were used to evaluate the performance of the developed nanobiocatalysts. Tygon tubing was used to link the microreactors to an automated SP200 Series syringe pump (World Precision Instruments, Hertfordshire, UK). The outer surface of the microreactors was placed on a magnet to stabilize the magnetic nanobiocatalyst on its inner surface (Fig. 1).

Optimization of the microreactor focused on two parameters: the quantity of biocatalyst and the flow rate. Cel@MbG was employed as the model system throughout this process. Firstly, 0.25 mg or 0.50 mg of cel@MbG were loaded in the microreactor, and the productivity was estimated. Secondly, four flow rates (2, 4, 8, and 16 μL min^−1^) were tested to find the ideal one. A 1% (w/v) CMC solution was pumped through the microreactor at 60 °C, and the effluent was collected at the outlet to measure glucose production (g_glucose_ L^−1^ h^−1^) using the DNSA assay.

The optimized parameters (0.50 mg biocatalyst, 4 μL min^−1^ flow rate) were applied to the bi-enzymatic biocatalyst. The operational stability of cel/bgl@MbG was evaluated for the hydrolysis of 1% w/v CMC for a 100 minute reaction time, at 60 °C. As an indicator of the enzyme usage efficiency and process performance, the modified turnover frequency (TOF) was defined using the following equation:^30^3
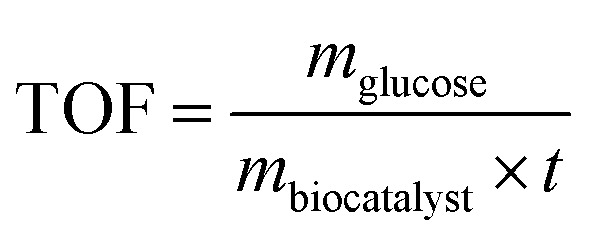
where *m*_glucose_ is the amount of glucose produced from the enzymatic hydrolysis (mg), *m*_biocatalyst_ is the amount of biocatalyst used in the enzymatic hydrolysis (mg), and *t* is the total time of enzymatic hydrolysis (*h*).

### 2.12. Statistical analysis

Each experiment was performed with three measurements taken per experiment. Results are presented as the mean ± standard deviation (SD). Statistical analysis for differences among groups (more than two) was conducted using one-way ANOVA followed by Tukey's multiple comparison tests, or two-way ANOVA followed by Tukey's test. A *t*-test was used to compare the two groups. Statistical significance was set at *p* < 0.05. Analyses were performed using IBM SPSS Statistics version 21 (SPSS Inc.).

## 3. Results

### 3.1. Synthetic route for MbG

**Fig. 2:**
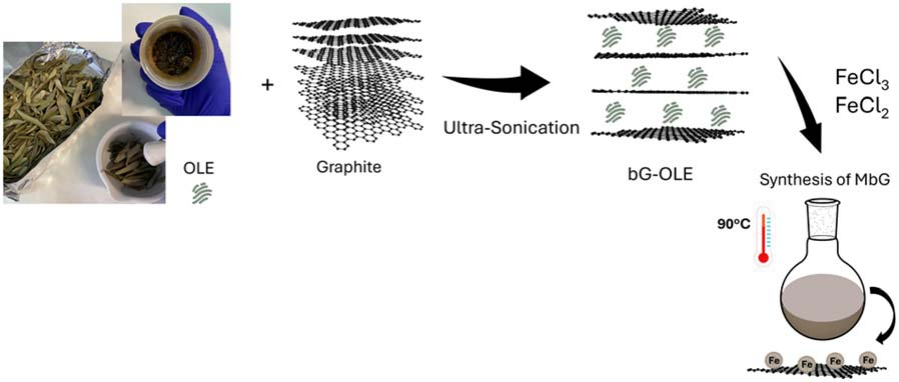
Depiction of the preparative steps toward the synthesis of MbG.

In this work, we describe the use of OLE as both the exfoliation agent of graphite and the reducing agent for the *in situ* formulation of MNPs on graphitic sheets towards the synthesis of magnetic bio-Graphene nanohybrid (Fig. 2). The phytochemical analysis of OLE reveals a significant amount of bioactive compounds, particularly phenolic compounds, proteins, and reducing sugars. OLE is particularly rich in polyphenols such as oleuropein and hydroxytyrosol. In our study, the TPC of OLE was recorded at 118.0 ± 1.5 mg caffeic acid equivalents (CAE) g^−1^ dry extract (DE). This result is consistent with other works for aqueous olive leaf extracts, highlighting the rich antioxidant properties of OLE.^34–37^ Additionally, the biochemical composition of OLE was tested by measuring the total protein content with the Bradford assay.^38^ The results showed that the total protein content in OLE was recorded at 17.0 ± 0.5 mg bovine serum albumin equivalents (BSAE) g^−1^ DE. This may also play a role in stabilizing the nanoparticles through steric hindrance and electrostatic interactions. Several studies have reported protein concentrations ranging from approximately 5 to 55 mg g^−1^ DE, depending on the solvents used for extraction and the extraction method.^39,40^

Furthermore, the analysis of total reducing sugars indicates the presence of carbohydrates that may contribute to the extract's functional properties.^33^ In our study, the total reducing sugars were measured at 140.7 ± 0.9 mg glucose g^−1^ DE, revealing the enhanced reducing capacity of OLE, which contributes to the efficient formation of nanoparticles while preventing their agglomeration.^39^ Collectively, these components underscore the potential of OLE, including its rich phytochemical content that can be utilized as an efficient reducing and stabilizing agent for the synthesis of magnetic nanohybrids.

Initially, the total content of OLE incorporated into the graphitic flakes during the synthesis of bio-Graphene was calculated at 27.8 ± 2.15%, using eqn (S1), described in the SI. After that, bG-OLE was utilized as the starting material for the synthesis of MbG. The optimization of synthesizing MbG involved adjusting the concentration of OLE (in bG-OLE) as the reducing factor and the ratio of the ferrous precursors, as described in the SI. The UV-Vis spectra showed that the ideal OLE concentration and FeCl_2_ : FeCl_3_ ratios were 0.35 mg mL^−1^ and 1 : 3, respectively (Fig. S1). The optimized material exhibited magnetic properties after applying an external magnetic field (Fig. S2). Moreover, AFM images reveal the successful exfoliation of graphite into graphene and the presence of MNPs on the surface of graphene. According to the AFM images, bG-OLE and MbG have a thickness of approximately 0.9 nm (Fig. S3). Generally, exfoliated materials are characterized by high specific surface area, enabling spatial organization of multi-enzyme systems and enzymes with high molecular weights.^41,42^ In previous works, bio-Graphene, a protein-exfoliated carbon nanomaterial, exhibited high enzyme loading in the co-immobilization of enzymes, structural stability of the immobilized enzymes, and effective realization of cascade reactions, rendering it an ideal green support.^10,43^ Moreover, the enrichment of bio-Graphene with nanoparticles can offer advantages in its final use, such as the easy separation from the reaction mixture by applying an external magnetic field, and the overall handling process of the arrangement of nanobiocatalysts.^23,24^

### 3.2. Characterization of MbG

#### 3.2.1. X-ray diffraction

**Fig. 3:**
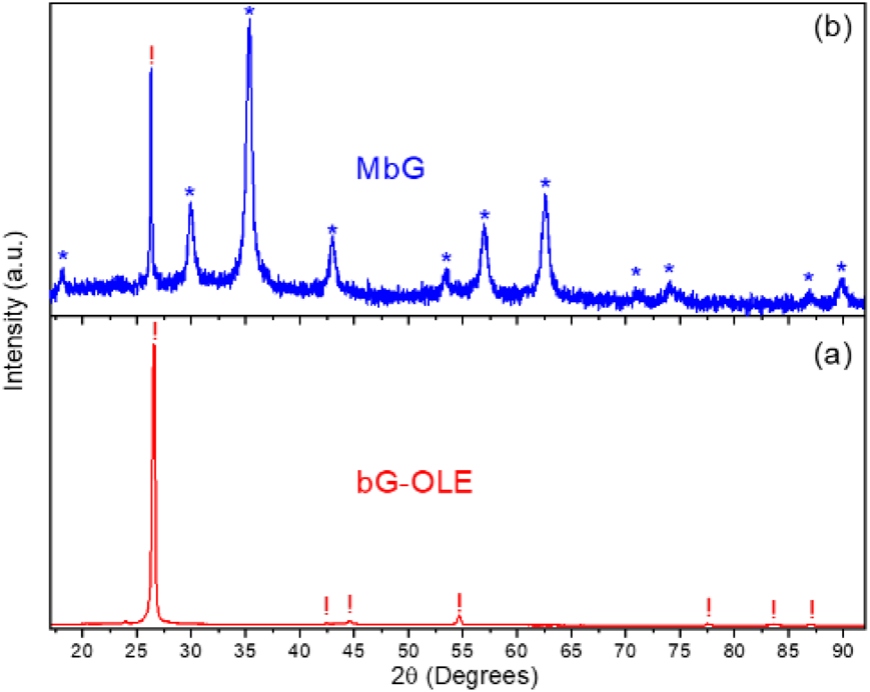
XRD diagrams of the bG-OLE (a) and MbG (b) samples. The crystalline phases in the samples are exhibited by the respective different symbols denoting the angular positions of the main diffraction peaks of magnetite-maghemite (*) and graphite (!).

The XRD diagrams of the bG-OLE and MbG samples are presented in Fig. 3. For the bG-OLE sample (Fig. 3a), the main diffraction peaks of the hexagonal graphite structure at 26.6 (002), 42.4 (100), 44.6 (101), 54.7 (004), 77.5 (110), 83.6 (112), and 87.1 (006) degrees 2*θ* (ICDD PDF 04-006-5764) are observed. The formation of a well-shaped and narrow main diffraction peak at 26.6 2*θ* indicates the high crystallinity of the graphite phase, which, combined with OLE, comprises the starting synthesis material.

On the other hand, the XRD pattern of the MbG sample shown in Fig. 3b, exhibits combined structural features, as indicated by the presence of the characteristic sharp diffraction peak of graphite at 26.6 (002) degrees 2*θ* (denoted with the ! symbol), and those of a spinel-type iron oxide magnetite-maghemite phase at 18.3 (111), 30.1 (220), 35.4 (311), 43.0 (400), 53.4 (422), 56.9 (511), 62.5 (440), 70.9 (620), 73.9 (533), 86.6 (642), and 89.6 (731) 2*θ* (ICCD PDF 01-089-0688) (denoted by the * symbols), which are however quite broader in shape compared to the graphite peak, indicating a decreased particle size for this phase in the sample. Due to this broadening, a safe conclusion on the nature of the exact spinel-type iron oxide phase is quite difficult to draw from XRD patterns alone,^44^ as magnetite and maghemite are indistinguishable under these conditions. Nevertheless, an estimation of the average MNP domain size 〈*D*〉, based on the most resolvable widths of the main diffraction peaks from this pattern, was performed by using the Scherrer formula,^45^ providing 〈*D*〉 ≈ 15 nm. This result indicates the ability of this synthesis method to produce very small MNPs developed on the surface of MbG.

#### 3.2.2. X-ray photoelectron spectroscopy

**Fig. 4:**
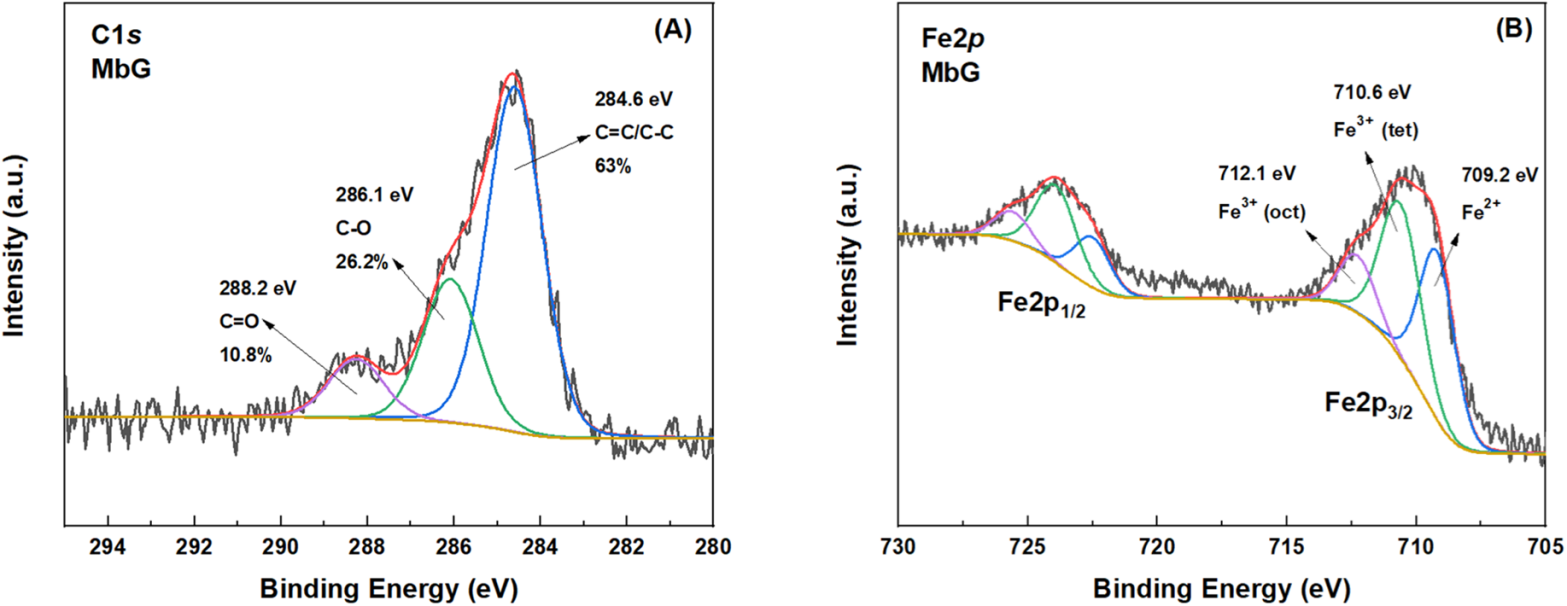
XPS spectrum of (A) the C 1s and (B) the Fe 2p core level of MbG.

XPS analysis of MbG exhibits the surface functionalization of the material. The C 1s of the initial material is presented in Fig. 4A. In this spectrum, it can be detected that the main C=C/C–C peak of MbG is around 63%. Two fitted peaks at 286.1 and 288.2 eV, corresponding to C–O and C=O bonds, respectively, can be attributed to the presence of OLE ingredients during the exfoliation procedure. Finally, the presence of iron oxide particles is confirmed by the Fe 2p photoelectron peak (Fig. 4B). From high-resolution spectra of iron 2p, three different chemical states of iron can be observed. The first peak, centered at 709.2 eV, is attributed to FeO compounds. The second peak, located at 710.5 eV, is attributed to Fe_3_O_4_ functional groups of Fe^3+^ (oct). Finally, the peak centered at 712.1 eV is due to Fe^3+^ (tet) compounds.^46^

#### 3.2.3. ^57^Fe Mössbauer spectroscopy

**Fig. 5:**
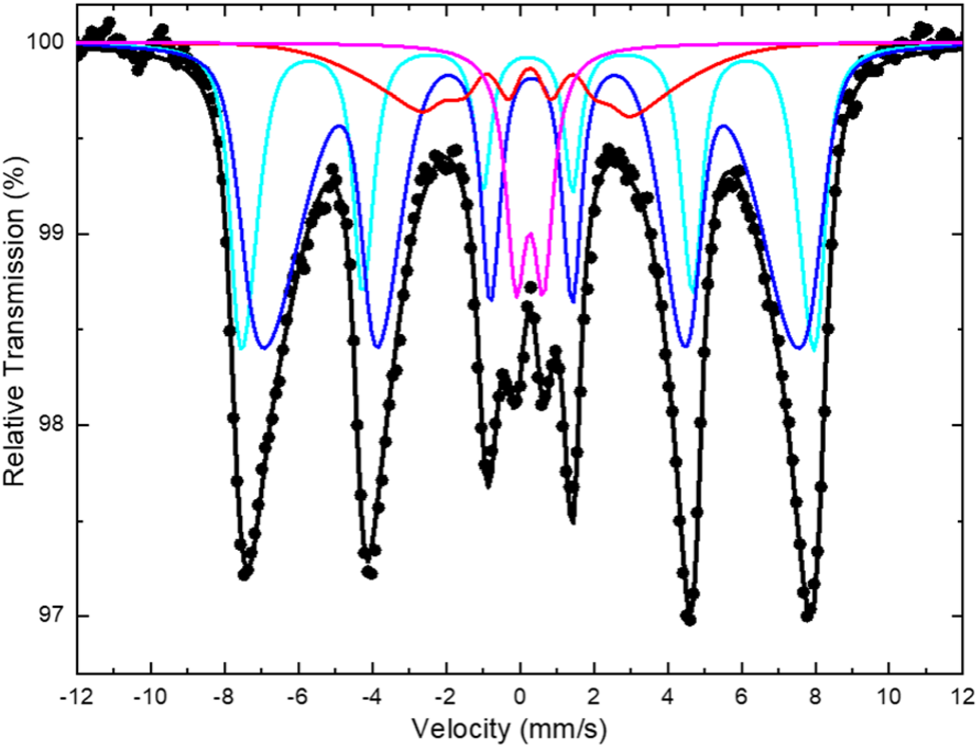
^57^Fe Mössbauer spectrum of the MbG sample collected at room temperature. The points correspond to the experimental data, and the continuous lines to the components used to fit the spectrum.

^57^Fe Mössbauer spectroscopy was used to determine in detail the nature and properties of the iron-bearing phases in the MbG sample. Fig. 5 displays the ^57^Fe Mössbauer spectrum of the MbG sample recorded at RT. The spectrum exhibits a prominent, broad, and asymmetric magnetically split contribution combined with an inferior central quadrupole split contribution. Moreover, a further “internal structure” of the outer-most resonant lines of the spectrum seems to be present, which is comprised of a sharper outer part and a broader inner part, indicating that two individual magnetically split component (M1 and M2) are necessary to be used to fit this part of the spectrum adequately. For these magnetically split components, an asymmetric Gaussian-type distribution (Δ*B*_hf_) of their hyperfine magnetic field (*B*_hf_) values around a central *B*^C^_hf_ value was allowed to accommodate the asymmetric broadening of the resonant lines.^32^ Moreover, to fit the inner part of the spectrum sufficiently, we used an additional magnetically split component with collapsing *B*_hf_ characteristics (M3), along with a minor central quadrupole split component (D1). The Mössbauer parameters values resulting from the best fit of this spectrum are listed in Table 1. The two prominent magnetically split components M1 and M2, colored cyan (Fe^3+^/Fe_3−*x*_O_4_) and blue (Fe^2·*ν*+^/Fe_3−*x*_O_4_) respectively in Fig. 5, acquire IS and quadrupole shift (2*ε*) values that are close to those found for the Fe^3+^ and Fe^2.5+^ ions on the A-tetrahedral and B-octahedral sites of magnetite. However, the IS value of the Fe^3+^/Fe_3−*x*_O_4_ component is shifted higher, and the IS value of the Fe^2·*ν*+^/Fe_3−*x*_O_4_ component is shifted lower than those found for the Fe^3+^ and Fe^2.5+^ components of the stoichiometric magnetite spectrum at RT, respectively.^47^ Considering that the expected iron-bearing magnetic phases in this sample are the spinel-type iron oxides magnetite and maghemite, as well as their nanostructured nature of these phases in it, we can ascribe these shifts, and consequently the iron oxide phase represented by these two major components in the Mössbauer spectrum, to an oxidized non-stoichiometric magnetite phase of a Fe_3−*x*_O_4_ average composition, with 0 < *x* < 1/3;^48–50^ we note here that the extreme cases of this spinel-type iron oxide composition are non-oxidized magnetite (Fe_3_O_4_, *x* = 0) and fully oxidized maghemite (Fe_2.67_O_4_ or γ-Fe_2_O_3_, *x* = 1/3). Evidence for the presence of this average partially oxidized Fe_3−*x*_O_4_ phase is the IS values of the two major Fe^3+^ and Fe^2·*ν*+^ components (with 2.5 < 2·*ν* < 3.0), which both approach the IS value of maghemite from different origins, as described above. The magnetically split component with collapsing *B*_hf_ characteristics (M3) and the quadrupole split component D1 acquire IS, 2*ε*, and QS values that are close to those expected for maghemite MNPs, which experience fast and very fast superparamagnetic (SPM) relaxation due to their small and very small particle sizes, respectively.^51^ From the above results, we can identify the stoichiometry and nature of the iron-bearing phases in the MNPs assembly of the MbG sample. In particular, the larger MNPs are ascribed to a spinel-type iron oxide Fe_3−*x*_O_4_ partially oxidized-magnetite phase. At this temperature (300 K), the sizes of these partially oxidized-magnetite MNPs developed on the surfaces of the bG-OLE growth platforms during the synthesis of the MbG sample, surpass the SPM size-limit, above which the SPM relaxation time *τ* is higher relative to the characteristic ^57^Fe Mössbauer spectroscopy measuring time *τ*_MS_ ∼10^−8^ s, and thus these MNPs are represented by the clearly (although broadened) magnetically split Fe^3+^/Fe_3−*x*_O_4_ and Fe^2·*ν*+^/Fe_3−*x*_O_4_ components in the corresponding spectrum.^48,52,53^ There are, however, smaller MNPs in the MbG sample, which acquire the fully oxidized spinel-type iron oxide phase of maghemite and are represented by the M3 and D1 components. The former component represents the part of the MNPs assembly for which their sizes are small enough to present *τ* values that are comparable to *τ*_MS_, and their distribution around *τ*_MS_ is the reason for the appearance of the collapsing *B*_hf_ characteristics. The latter component represents the MNPs with the smallest sizes in the corresponding assembly, for which *τ* is lower than *τ*_MS_, and this is the reason for the appearance of the SPM characteristics. It is worth noting here that the shift in stoichiometry is accompanied by a shift in the size observed for the MNPs of the assembly. This result can be attributed to the nature of the spinel-type iron oxide magnetite-maghemite phases resulting from the relative synthesis of the MbG sample, and the relation between them. The smaller MNPs, due to their reduced sizes, are more susceptible to oxidation and are fully oxidized during the synthesis procedure, while the larger MNPs should oxidize only partially and mainly on their surfaces. Thus, the stoichiometry of the assembly moves from a Fe_3−*x*_O_4_ partially oxidized-magnetite phase for the larger MNPs to a fully oxidized-maghemite γ-Fe_2_O_3_ phase for the smaller MNPs.^32,48,51^

**Table 1:** Mössbauer hyperfine parameters resulting from the best fit of the corresponding spectrum of the sample shown in Fig. 5 a

IS, mm s^−1^	*Γ*/2, mm s^−1^	QS or 2*ε*, mm s^−1^	*B* ^C^_hf_, kOe	Δ*B*_hf_ or Δ*B*_hf_ < *B*^C^_hf_/Δ*B*_hf_ > *B*^C^_hf_, kOe	Area, %	Component/color	Assignment
0.32	0.25	0.00	493	12/1	27	M1/cyan	Fe^3+^/Fe_3−*x*_O_4_
0.41	0.25	0.00	464	31/10	53	M2/blue	Fe^2·*ν*+^/Fe_3−*x*_O_4_
0.33	0.25	−0.09	170	6/52	11	M3/red	Collapsing *B*_hf_ γ-Fe_2_O_3_
0.37	0.33	0.74	0	0	9	D1/magenta	Superparamagnetic γ-Fe_2_O_3_

a IS: isomer shift (given relative to α-Fe at 300 K); *Γ*/2: the half-line width; QS: the quadrupole splitting; 2*ε*: the quadrupole shift; *B*^C^_hf_: the central value of the hyperfine magnetic field; Δ*B*_hf_ is the total spreading (Gaussian-type) of the *B*_hf_ values around the central *B*^C^_hf_ value; AA: the relative spectral absorption area of each component used to fit the spectrum. For asymmetric *B*_hf_ spreading around *B*^C^_hf_ the two Δ*B*_hf_ figures denote values lower/higher than *B*^C^_hf_. Typical errors are ±0.02 mm s^−1^ for IS, *Γ*/2, 2*ε*, and QS, ±3 kOe for *B*^C^_hf_, and ±3% for AA.

#### 3.2.4. Vibrating-sample magnetometry

**Fig. 6:**
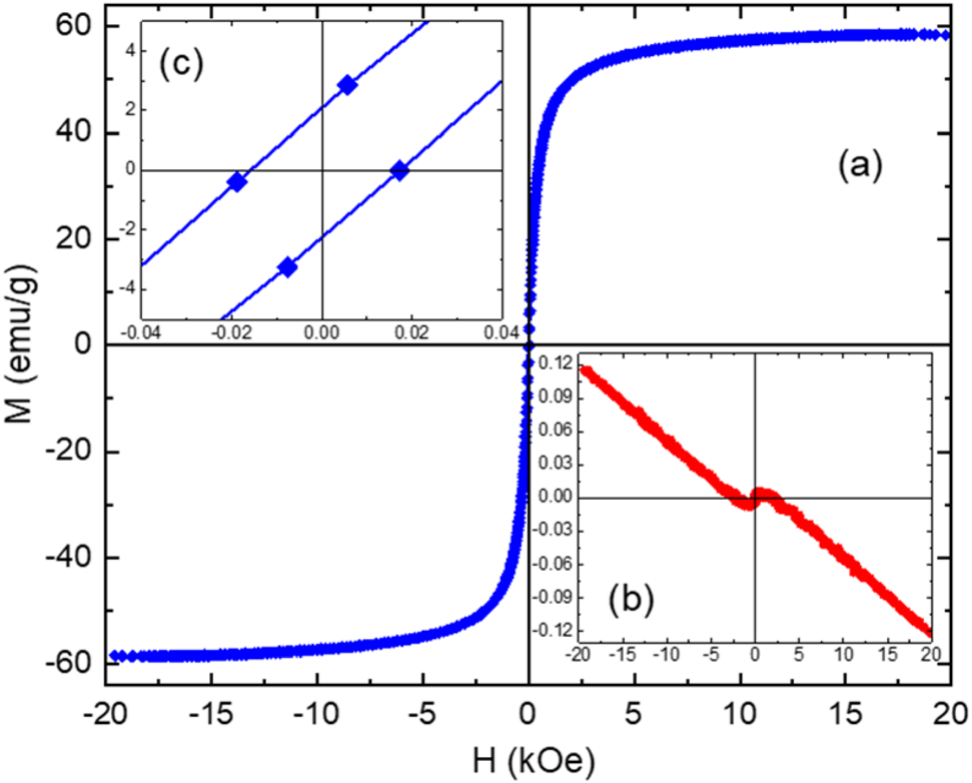
Room temperature magnetization *vs.* applied magnetic field isothermal loops of the MbG (a) and bG-OLE (b) samples. The upper left inset (c) shows details of the MbG sample's loop characteristics around zero applied magnetic field.

The magnetic properties of both bG-OLE and MbG samples are revealed by their *M vs. H* measurements conducted at RT. The *M vs. H* loop of the MbG sample shown in Fig. 6(a) demonstrates distinct ferrimagnetic characteristics typical of the magnetite-maghemite nanostructured phases, with very small hysteresis and a coercivity of approximately 17 Oe (Table 2). A slight tendency for incomplete saturation of *M* at high *H* values indicates that the dominant ferrimagnetic contribution is accompanied by an additional SPM relaxation at this temperature. These features emerge from the particle size distribution of the magnetite-maghemite MNPs, as evident also by the XRD and Mössbauer measurements.^53–55^ In particular, the larger and/or strongly interacting MNPs developed on the surfaces of the supporting bG-OLE material dominate the total magnetically blocked ferrimagnetic behavior of the MbG sample, while the smaller and/or weakly interacting MNPs are identified by the lack of *M* values saturation, presenting the SPM characteristics. The *M vs. H* loop of the bG-OLE sample shown in Fig. 6(b) exhibits clear diamagnetic characteristics at high fields, which are typical for the graphitic-type structure present in this sample, while a small bending in the signal around zero *H* values denotes the presence of a minor paramagnetic residual.

**Table 2:** Parameters of the magnetic properties derived from the isothermal loops of Fig. 6

Sample	*T* (K)	*M*_max_ (emu g^−1^)	*M*_min_ (emu g^−1^)	*M*_R+_ (emu g^−1^)	*M*_R−_ (emu g^−1^)	*H*_C+_ (Oe)	*H*_C−_ (Oe)
bG-OLE	300	0.12	−0.12	0	0	0	0
MbG		58.5	−58.7	2.2	−2.6	18	−16

### 3.3. Immobilization of biocatalysts on MbG

#### 3.3.1. Optimization of the immobilization conditions

**Fig. 7:**
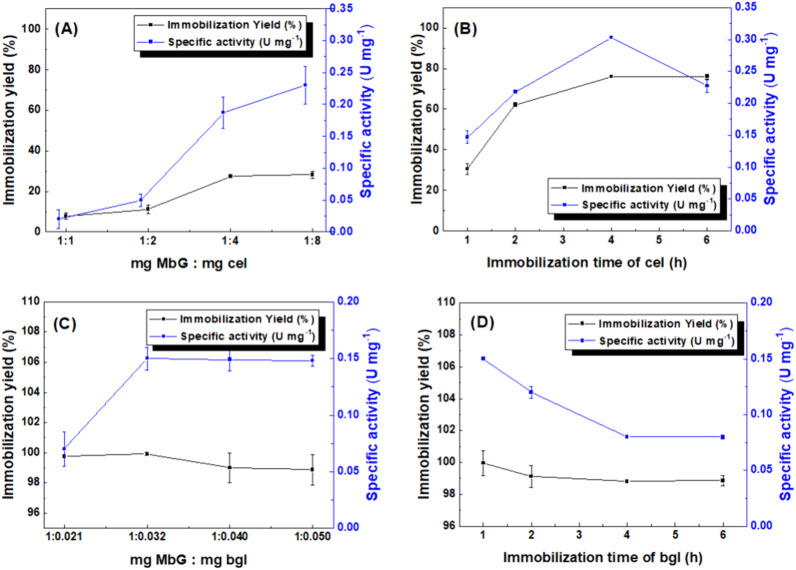
The effects of immobilization conditions on the activity (blue) and immobilization yield (black) of nanobiocatalysts. Effect of enzyme-to-nanosupport mass ratio on immobilized cel (A) and bgl (C), and effect of immobilization incubation time on cel@MbG (B) and bgl@MbG (D). All analyses were conducted in triplicate, and the results are shown with the associated standard deviations (Tables S1–S4) (in some cases, the standard deviation was <0.005 for specific activity and <1% for immobilization yield; therefore, the error bars are not visible).

**Fig. 8:**
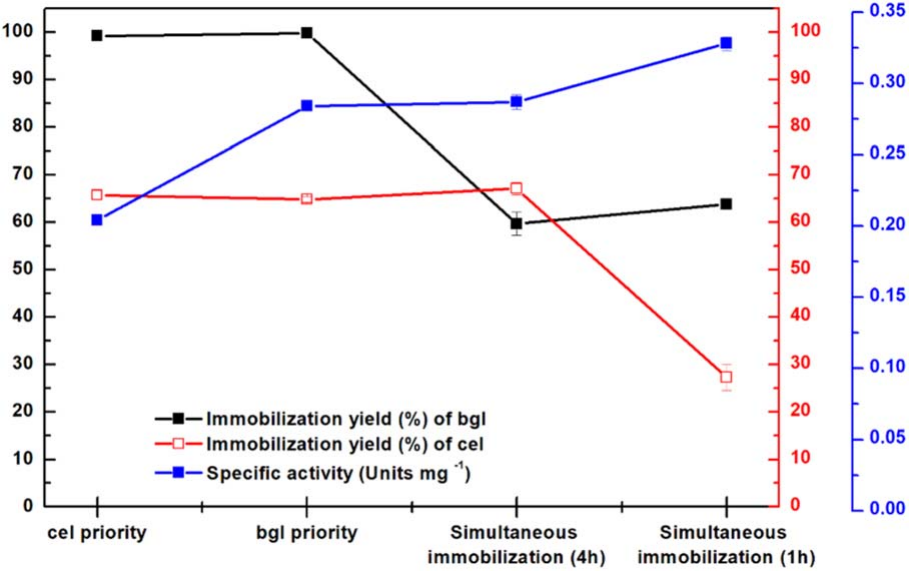
Effect of simultaneous and sequential immobilization on the activity (blue) of the co-immobilized nanobiocatalyst, the immobilization yield of bgl (black), and the immobilization yield of cel (red). All analyses were conducted in triplicate, and the results are shown with the associated standard deviations (Table S5) (in some cases, the standard deviation was <0.005 for specific activity and <1% for immobilization yield; therefore, the error bars are not visible).

The synthesized MbG was used as a matrix for the physical adsorption (individual immobilization and co-immobilization) of cel and bgl. Two parameters, the enzyme-to-carrier mass ratio and the immobilization incubation time, affected the immobilization yields and specific activities of the individually immobilized enzymes, and the results are presented in Fig. 7. Fig. 7A and C illustrate the immobilization yield and hydrolytic activity of immobilized cel and bgl in correlation with the enzyme-to-support mass ratio. It can be assumed that the conformation of enzymes is altered due to overlapping coverage of partially active sites by adjacent enzymes, leading to decreased activity, as mentioned before.^56,57^ In addition, another parameter was tested for optimizing the nanobiocatalysts, namely, the incubation time of the enzymes with the nanocarrier during the immobilization procedure. Fig. 7B shows that as the immobilization incubation time increased, the activity and immobilization yield of cel@MbG increased correspondingly. However, after 4 h of incubation, a decline in specific activity was observed. In the case of bgl@MbG, the immobilization incubation time did not affect the immobilization yield, however, the specific enzymatic activity declined after 1 h incubation (Fig. 7D). These results are in agreement with previously reported studies, probably because when the enzymes interact with the nanocarrier for a long time, too many enzyme molecules could be bound onto the surface of the magnetic carrier, changing the configuration of enzymes and leading to a decrease in their activity.^58–60^ In the next step, sequential and simultaneous co-immobilization of cel and bgl was employed to enhance the enzymes' synergistic action in the CMC hydrolysis reaction. Based on the optimized immobilization incubation time of each enzyme (1 h for bgl and 4 h for cel), four versions of co-immobilized biocatalysts were formed (2 simultaneous and 2 sequential) (Fig. 8). The results showed that the bi-enzymatic system composed after simultaneous immobilization exhibited significantly higher activity (*p* < 0.0001) than those obtained by 1 h sequential immobilization. Simultaneous immobilization is particularly advantageous in applications requiring synergistic effects from co-immobilized biomolecules, such as in cascade enzymatic reactions or multi-enzyme systems, as mentioned before.^61^

In our study, in the 1 h simultaneous co-immobilization, the immobilization yields of cel and bgl were low, following a competition effect for their position in MbG; however, the highest specific activity was recorded (∼0.33 units mg^−1^, *p* < 0.0001). This can be attributed to the efficient disposal of the biocatalysts in the matrix in contrast to the other versions.^56,62^ In addition, the specific activity of the bi-enzymatic nanobiocatalyst surpassed that of the individually immobilized cel (0.15 units mg^−1^) after 1 h of incubation, highlighting the positive synergistic effect of bgl in the hydrolytic activity.^28^ Table S6 summarizes the activity of various cellulase immobilization systems, highlighting the influence of immobilization method and carrier on cellulase catalytic activity and operational performance. In the 4 h simultaneous co-immobilization, the prolonged incubation time probably allowed the overpacking of enzymes on the nanomaterial's surface, preventing the substrate's easy access to the enzyme and explaining the decrease in specific activity.^58,60^ On the other hand, the biomolecules were immobilized one after another in successive immobilization. In Fig. 8, although the immobilization yields were high in both cases (cel priority and bgl priority), the specific activities were not ideal. This indicates that the first enzyme attached to MbG occupied a large surface, overlapping the next-in-line enzyme and reducing its activity, as previously specified.^63,64^ Simultaneous co-immobilization often yields higher activity than sequential immobilization due to optimal enzyme proximity, which facilitates rapid transfer of intermediates and reduces diffusion limitations between enzymes in cascade reactions.^65^ Studies show that co-immobilizing enzymes in close proximity on a shared support mimic natural multi-enzyme complexes, supporting efficient cascade catalysis and higher specific activity.^66,67^

According to the above results, simultaneous co-immobilization at 1 h incubation time seems to be the most effective bi-enzymatic system; thus, this biocatalyst was further characterized biochemically and morphologically, and it will be referred to as cel/bgl@MbG from now on.

#### 3.3.2. Spectroscopic characterization of the bi-enzymatic nanobiocatalyst

Characterizing the bi-enzymatic nanobiocatalyst using FTIR and XPS spectroscopies provides valuable insights into the structural and chemical properties of the immobilized enzymes on nanomaterials. FTIR analysis confirmed the successful immobilization of enzymes on MbG (Fig. S4A). The presence of cel and bgl on MbG was validated by the appearance of the amide II (1556 cm^−1^) and amide III (1406 cm^−1^) bands, corresponding to the C–N stretching and N–H bending vibrations of the amide bonds in the protein chains.^10,59^ The bands at 628 cm^−1^ correspond to Fe–O elongation from MNPs conjugated on the surface of graphene flakes.^68,69^ The weak band of MbG at 1648 cm^−1^ (ascribed to the presence of OLE) has strengthened and shifted to 1650 cm^−1^ (amide I) after enzyme immobilization, indicative of the C=O stretching vibrations derived from the peptide bonds of the proteins.^31^ Finally, the band at 3420 cm^−1^ arises from O–H stretching vibrations.^3^

For the XPS analysis of cel/bgl@MbG, the C 1s high-resolution photoelectron peak was applied (Fig. S4B). In detail, the C 1s spectrum revealed the oxygen functionalities derived from the enzyme structure and the C=C peak from MbG. The increased percentage of oxygen functionalities compared to the initial material can be ascribed to the successful immobilization of cel and bgl. In detail, the spectrum revealed the C–OH, C–O–C, and C=O functional groups resulting from the enzyme's interaction with the surface of MbG. A new C–O–C peak appeared after the co-immobilization, which can be attributed to the presence of enzymes on the surface of MbG. Furthermore, the C=O groups, which correspond to carbonyl groups in enzyme peptide bonds, increased from 10.2% in MbG to 17.4% after co-immobilization of cel and bgl.

#### 3.3.3. Biocatalytic characterization of the bi-enzymatic system

The most fundamental and significant factors influencing the catalytic activity of an enzyme are the pH and temperature of the enzymatic reaction. A proper pH value promotes the dissociation state in which the substrate and the intermediate complex are most suitable for the enzymatic reaction, and preserves the structure of the enzyme's binding site, and the important cleavable group of the catalytic site in an ideal condition.^70,71^ In addition, the temperature of the catalytic process can influence its equilibrium and the activation of the substrate molecule. Furthermore, the immobilization technique and the carrier can affect the enzyme's ideal pH and temperature.^72,73^

**Fig. 9:**
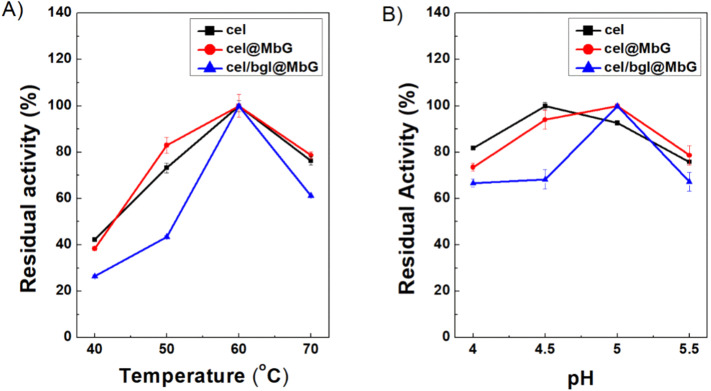
Effect of (A) temperature and (B) pH on the activity of free cel, cel@MbG, and cel/bgl@MbG. One hundred percent indicates the highest activity exhibited each time, either by the free enzyme or the nanobiocatalyst tested. All analyses were conducted in triplicate, and the results are shown with the associated standard deviations (Tables S7 and S8) (in some cases, the standard deviation was <1%; therefore, the error bars are not visible).

As seen in Fig. 9A and S5A, the optimal temperature of cel@MbG and bgl@MbG was 60 °C and 70 °C, respectively. Although the relative activity of free and immobilized cel decreased after 60 °C, the decline was not significant between them (*p* > 0.05). In the case of bgl@MbG (Fig. S5A), the residual activity increases while the temperature shifts to 70 °C (*p* < 0.0001). It is known that bgl is a recombinant β-glucosidase derived from the hyperthermophilic bacterium *Thermotoga maritima*, explaining its stability at high temperatures.^10,74^

In addition, the support effectively protects the enzyme molecules at higher temperatures, as has been previously reported.^75^ The co-immobilized cel/bgl@MbG followed the same behavior as the individually immobilized cellulase and reached maximum activity at 60 °C. At temperatures above and below 60 °C, the catalytic activity of cel/bgl@MbG decreased more than cel@MbG. For instance, at 70 °C, the residual activity of cel/bgl@MbG is 61.3%, in contrast to the individually immobilized form, where the residual activity is 78.6%. This result could be correlated with the protein molecules attached to the surface of MbG; the immobilization yield of cel in the cel@MbG system is ∼2.7-fold higher than in the cel/bgl@MbG system (as exported from Fig. 7 and 8), indicating that more cel molecules have been immobilized in the first case, and thus, maximizing the structural stabilization of the protein molecules against thermal denaturation.^76^

According to Fig. 9B and S5B, cel@MbG and bgl@MbG showed maximum activity at pH 5.0 and 5.5, respectively, following the optimum pH of the free enzymes. Similar results have been reported before,^60,75,77^ indicating that no significant changes occur in the immobilized enzyme's microenvironment upon immobilization. The relative enzymatic activity of bgl@MbG was significantly higher (*p* < 0.0001) than that of the free enzyme at pH 4.0 and 4.5 (Fig. S5B). This corresponds to previously published results reporting that increased pH stability in acidic conditions for immobilized enzyme systems is related to secondary interactions between the enzyme molecules and supports.^74,78^ In the case of cel, the maximum activity of free cel was measured at pH 4.5, and that of cel@MbG was found at pH 5.0; however, no significant difference was recorded (*p* > 0.05). In the case of cel/bgl@MbG, the optimal pH was 5.0 (*p* < 0.0001). This could be attributed to the coupling of the enzyme with MbG, revealing interactions that may alter the local microenvironment around the enzyme by affecting its pH stability and potentially stabilizing the enzyme's structure at a higher or lower pH, as mentioned before.^79–81^ By combining the ideal temperature and pH of free and immobilized (both individually and co-immobilized) enzymes, the optimal temperature and pH of the co-immobilized form were found to be consistent with the behavior of cel@MbG and free cel, revealing the dominant role of cel in the activity of the biocatalytic system.

**Fig. 10:**
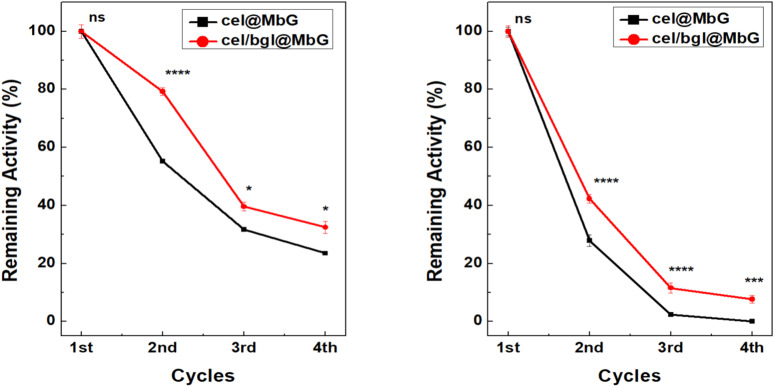
Reusability of the immobilized nanobiocatalytic systems for the hydrolysis of CMC (A) and Avicel (B). All analyses were conducted in triplicate, and the results are shown with the associated standard deviations (Tables S9 and S10) (in some cases, the standard deviation was <1%; therefore, the error bars are not visible). Significant differences between the two nanobiocatalytic systems at each cycle are represented by asterisks (*p < 0.05; ***p < 0.01; ****p < 0.001; ns = not significant), using two-way ANOVA analysis.

Using magnetic carriers such as MbG offers the advantage of easy separation, recovery, and reusability of the immobilized enzymes by applying an external magnetic field.^28,82^ In this work, the reusability of the mono- and bi-enzymatic system was investigated, following the hydrolysis of CMC and Avicel PH101. Results highlight that the supported nanobiocatalysts hydrolyzed CMC and Avicel PH101, up to 4 consecutive reaction cycles (Fig. 10). The cel/bgl@MbG biocatalyst retained about 32.5 and 8% of its initial activity after four cycles of CMC and Avicel hydrolysis, respectively, while the residual activity of cel@MbG was 23.5% for CMC and was nearly depleted for Avicel. Overall, the reusability of the bi-enzymatic system was superior to the mono-enzymatic system until the 4th cycle, demonstrating the synergistic activity of bgl and cellulase, boosting glucose production.^83^ The simultaneous co-immobilization squeezed cel and bgl close together, thus decreasing the mass transfer restrictions of macromolecular substrates such as CMC and Avicel.^84^ Several authors have reported the operational stability of co-immobilized enzymatic systems.^72,85,86^ However, a significant loss of hydrolytic activity was observed after the second cycle. The sharp decrease in activity after the first catalytic cycle could be associated with the immobilization method. Physical adsorption is the simplest immobilization process, but it has certain drawbacks. One of them is the easy detachment of the support, which results in the leaching of the enzyme.^87^ To address this limitation, the introduction of covalent attachment methods could enhance the stability of the enzyme–support interaction. Such modifications would not only minimize leaching but also potentially extend the operational lifetime of the system.^68,88^

### 3.4. Application of the developed nanobiocatalysts in microreactor systems

#### 3.4.1. Optimization of the immobilized microreactor

The first step in optimizing a microreactor system is estimating the amount of immobilized enzyme based on the observed productivity. In this work, two different amounts of the model mono-enzymatic system cel@MbG were loaded in the microreactor and tested for their productivity. The results presented in Table 3 indicate that productivity is significantly enhanced 2.11 times (*p* < 0.05), with increasing loading of cel@MbG in the microreactor. As the immobilized enzyme load increases, the internal nanobiocatalyst-coated microreactor results in higher conversion rates.^89,90^ Higher amounts of immobilized enzyme were not tested due to the insolubility of the nanobiocatalyst. Therefore, the next experiments were carried out with 0.50 mg of the immobilized system.

**Table 3:** Effect of the amount of cel@MbG on the productivity of the microreactor

Biocatalyst's amount (mg)	Productivity (g_glucose_ L^−1^ h^−1^)
0.25	0.111 ± 0.025
0.50	0.223 ± 0.010

**Fig. 11:**
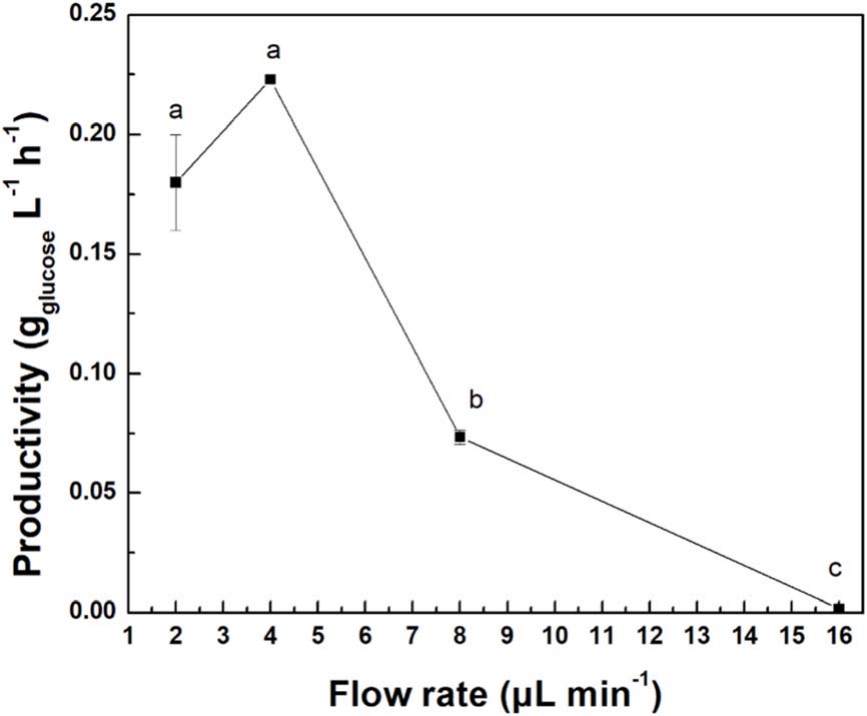
Effect of the flow rate on the productivity of cel@MbG microreactor. All analyses were conducted in triplicate, and the results are shown with the associated standard deviations (Table S11) (in some cases, the standard deviation was <0.001; therefore, the error bars are not visible). Significant differences (*p* < 0.05) among the different flow rates of the cel@MbG microreactor are represented by lowercase letters, using one-way ANOVA analysis.

Flow rate is another crucial parameter affecting enzymes' behavior in a microreactor's interior.^91^Fig. 11 presents the results from the flow rate study. At low flow rates (2 and 4 μL min^−1^), cel@MbG exhibits the highest productivity (0.18 and 0.22 g_glucose_ L^−1^ h^−1^, respectively). It can be inferred that increasing the flow rate significantly decreases productivity. This observation agrees with previous studies on microfluidic systems and is explained by considering the enzyme–substrate residence time.^92^ As the flow rate increases, the available time for enzyme–substrate interaction decreases, leading to lower conversion yields, as previously reported for a bgl continuous flow system.^93^ Although there is no significant difference between 2 and 4 μL min^−1^ flow rate (*p* > 0.05), the latter was selected to reduce the operational time of the procedure, avoiding possible temperature denaturation of the nanobiocatalyst.^94^

#### 3.4.2. Operational stability of the microreactor

Continuous operation using microreactors presents a key advantage over traditional batch systems by improving the overall cost-effectiveness of the process. The hydrolysis of CMC for evaluating operational stability is demonstrated in a flow microreactor system catalyzed by the optimal batch systems of mono- (cel@MbG) and bi-enzymatic (cel/bgl@MbG) nanobiocatalytic systems, and the productivity and TOF are presented in Table 4. The microreactor system of cel/bgl@MbG exhibited the highest productivity, which was 1.3 times higher than that of the microreactor of cel@MbG. Other publications have demonstrated similar outcomes, revealing the effectiveness of enzyme microreactors.^30,95^ The TOF of the bi-enzymatic microreactor was ∼3.6 times greater than the mono-enzymatic microfluidic system (*p* < 0.0001). It is interesting to note that the immobilization yield of cel in the cel/bgl@MbG nanobiocatalytic system is lower (∼2.7-fold) than that in the cel@MbG, meaning that fewer cel molecules are available to participate in the hydrolysis reaction, and thus, lower sufficient β-glucosidase activity towards glucose production. It can be concluded that the addition of bgl boosts glucose production by completing the final step of cellulose hydrolysis, highlighting the synergistic effect of the two enzymes on the reaction performance.^28,96^ Furthermore, the MbG created an ideal host for multi-enzyme immobilization, enhancing the simplicity and cost-effectiveness of microfluidic systems fabrication.^23,24^ The advantage of the prepared magnetic microreactors can be associated with their easy fabrication. Usually, microreactors need surface modification through chemical treatment to effectively host immobilized biomolecules. However, these treatments introduce harsh conditions that may compromise enzyme structure, which may affect both the immobilization yield and the catalytic performance of the enzyme.

**Table 4:** Comparative data of the operational stability of the microreactor and batch systems

Sample	Operational time (min)	Productivity (g_glucose_ L^−1^ h^−1^)	TOF (h^−1^)
Microreactor cel/bgl@MbG	100	0.300 ± 0.003	0.105 ± 0.001
Microreactor cel@MbG	100	0.223 ± 0.001	0.029 ± 0.001

Moreover, such chemical functionalization adds complexity, cost, and potential environmental concerns to the fabrication process. In our case, the microreactors were filled with nanobiocatalytic systems and stabilized with an external magnetic field, requiring additional steps in microreactor development. Nevertheless, further optimization of the microreactor design, setup, and operational conditions may be essential for achieving higher biocatalytic performance.

## 4. Conclusions

This study proposes the green synthesis of magnetic bio-Graphene (MbG) with olive leaf extract. The resulting magnetic material combines high surface area, inherent surface functionality, and magnetic recoverability. The potential of MbG for conjugating hydrolytic multi-enzymatic systems was subsequently evaluated through the immobilization of cellulase and β-glucosidase. Optimizing the immobilization of these enzymes on MbG resulted in a bi-enzymatic nanobiocatalyst with enhanced synergistic activity and improved reusability compared to mono-enzymatic systems. Both forms were successfully applied in continuous-flow microreactors with magnetic confinement, demonstrating enhanced productivity without harsh chemical modifications. This work illustrates the potential of green nanomaterials for multi-enzyme immobilization and efficient cellulose conversion, highlighting an approach that minimizes environmental impact while addressing challenges in enzyme immobilization and reactor design. To our knowledge, this is the first report of olive leaf extract-mediated green synthesis of magnetic bio-Graphene used for sustainable multi-enzymatic biocatalysis under batch and microfluidic conditions.

## Author contributions

Christina Alatzoglou: conceptualization, investigation, methodology, formal analysis, data curation, visualization, writing – original draft. Michaela Patila: conceptualization, methodology, formal analysis, data curation, visualization, validation, writing – original draft. Panagiotis G. Ziogas: investigation, methodology, formal analysis, data curation, visualization, writing – original draft. Anastasia Skonta: methodology, data curation, writing – review & editing. Despoina Politi: investigation. Konstantinos Spyrou: methodology, formal analysis, data curation, visualization, writing – original draft. Angela S. Kaloudi: investigation, methodology, visualization. Alexios P. Douvalis: methodology, formal analysis, data curation, validation, writing – review & editing. Dimitrios P. Gournis: methodology, validation, writing – review & editing. Haralambos Stamatis: conceptualization, methodology, validation, supervision, project administration, resources, funding acquisition, writing – review & editing.

## Conflicts of interest

The authors declare that they have no known competing financial interests or personal relationships that could have appeared to influence the work reported in this paper.

## Supplementary Material

RA-015-D5RA06271C-s001

## Data Availability

The data supporting this article have been included as part of the supplementary information (SI). Supplementary information is available. See DOI: https://doi.org/10.1039/d5ra06271c.
