# Supplementary material for: Green synthesis of magnetic bio-Graphene nanohybrid for the immobilization of hydrolytic enzymes towards sustainable bioconversion of cellulose
Source: RSC Adv. 2025 Oct 6;15(44):37194–208. doi: 10.1039/d5ra06271c (PMC12498520; doi:10.1039/d5ra06271c)
Supplement: RA-015-D5RA06271C-s001 [file RA-015-D5RA06271C-s001.pdf]

Supplementary Information (SI) for RSC Advances.
This journal is © The Royal Society of Chemistry 2025

Supplementary Information

Green synthesis of magnetic bio-Graphene nanohybrid for the immobilization of hydrolytic enzymes towards sustainable bioconversion of cellulose

Christina Alatzoglou [a], Michaela Patila [a], Panagiotis G. Ziogas [b], Anastasia Skonta [a], Despoina Politi [a], Konstantinos Spyrou [c], Angela S. Kaloudi [c], Alexios P. Douvalis [b], Dimitrios P. Gournis [d,e,] Haralambos Stamatis [a, *]

[a] Laboratory of Biotechnology, Department of Biological Applications and Technologies, University of Ioannina, Ioannina, Greece.

[b] Physics Department, University of Ioannina, 45110 Ioannina, Greece.

[c] Department of Materials Science & Engineering, University of Ioannina, 45110 Ioannina, Greece.

[d] School of Chemical and Environmental Engineering, Technical University of Crete, 73100 Chania, Greece.

[e] Institute of GeoEnergy, Foundation for Research and Technology-Hellas, 73100 Chania, Greece

* Correspondence: hstamati@uoi.gr (H.S.)

1. **Supplementary Materials and Methods**

1.1. Determination of total phenolic content, total protein content, and total reducing sugars

The total phenolic content of OLE was determined spectrophotometrically at 725 nm using the Folin-Ciocalteu assay according to [1], with slight modifications. In brief, in 0.164 mL of ddH_2O, appropriate aliquots of the extract solution in ddH_2O were added at a final concentration of 15 μg mL^{-1} and 30 μg mL^{-1}, followed by the addition of 10 μL of Follin-Ciocalteu reagent and 3-min incubation of the mixture at room temperature in the dark. After 3 min of incubation, 20 μL of a saturated solution of Na_2CO_3 (2% *w/v*) was added, and the final mixture was incubated for 1 h at room temperature in the dark. The absorbance of the samples was then measured at 725 nm. Blank samples were also prepared. Following the same procedure, a caffeic acid calibration curve was prepared at a final concentration range of 0–100 μg mL^{-1}. The results were expressed as equivalents of caffeic acid (mg) per gram of the extract. All experiments were performed thrice.

The total protein content of OLE was determined spectrophotometrically at 595 nm using the Bradford assay [2]. In brief, 200 μL of extract solution (in ddH_2O) at a final concentration of 1 mg mL^{-1}, followed by the addition of 800 μL of Bradford and 10-min incubation of the mixture at room temperature in the dark. The absorbance of the samples was then measured at 595 nm. Blank samples were also prepared. Following the same procedure, a bovine serum albumin (BSA) calibration curve of was prepared at a final concentration range of 0–20 μg mL^{-1}. The results were expressed as equivalents of BSA (mg) per gram of the extract. All experiments were performed thrice.

The total reducing sugar content of OLE was determined spectrophotometrically at 540 nm using the DNSA assay [3]. In brief, 250 μL of extract solution (in ddH_2O) at a final concentration of 3 mg mL^{-1}, followed by the addition of 250 μL of DNSA and 5 min incubation of the mixture at ~100 °C. After 2 mL of ddH_2O was added, the absorbance of the samples was measured at 540 nm. Blank samples were also prepared using 250 μL of ddH_2O instead of OLE. Following the same procedure, a glucose calibration curve was prepared at a final concentration range of 0–2 mg mL^{-1}. The results were expressed as equivalents of glucose (mg) per gram of the extract. All experiments were performed thrice.

1.2. Determination of OLE integration on bG

To determine the percentage of OLE incorporated on bG sheets, UV-Vis spectra (200-800 nm) were obtained in the supernatant during bG synthesis. A calibration curve of OLE was prepared at a final concentration range of 0–0.3 mg mL^{-1}. The percentage of the extract incorporated into bG was measured according to the following eqn (s1):

$$OLE\ integration\ on\ bG\ (\%) = \frac{[OLE]i - [OLE]s}{[OLE]i} \text{ x } 100 \qquad \text{(s1)}$$

where [OLE]i is the initial concentration of the extract and [OLE]s is the concentration of the remaining extract in the supernatant.

1.3. Optimization of the synthesis of magnetic bio-graphene

The synthetic procedure for magnetic bio-graphene (MbG) involved optimizing various parameters, including the salt ratio of iron precursors and the concentration of bio-graphene combined with olive leaf extract (bG-OLE). Different concentrations (0.18, 0.35, 0.70, and 1.4 mg mL^{-1}) of bG-OLE mixtures were used, as well as salt ratios (500:0, 0:500, 250:250, 135:365) of $FeCl_2$ to $FeCl_3$ (mg: mg) for the assessment of optimal stabilization and reducing capabilities of OLE in facilitating the synthesis process while ensuring effective interaction with iron nanoparticles. The results were evaluated by recording the UV-Vis spectra of the samples in the 200-800 nm region.

2. Supplementary Results, Figures and Tables

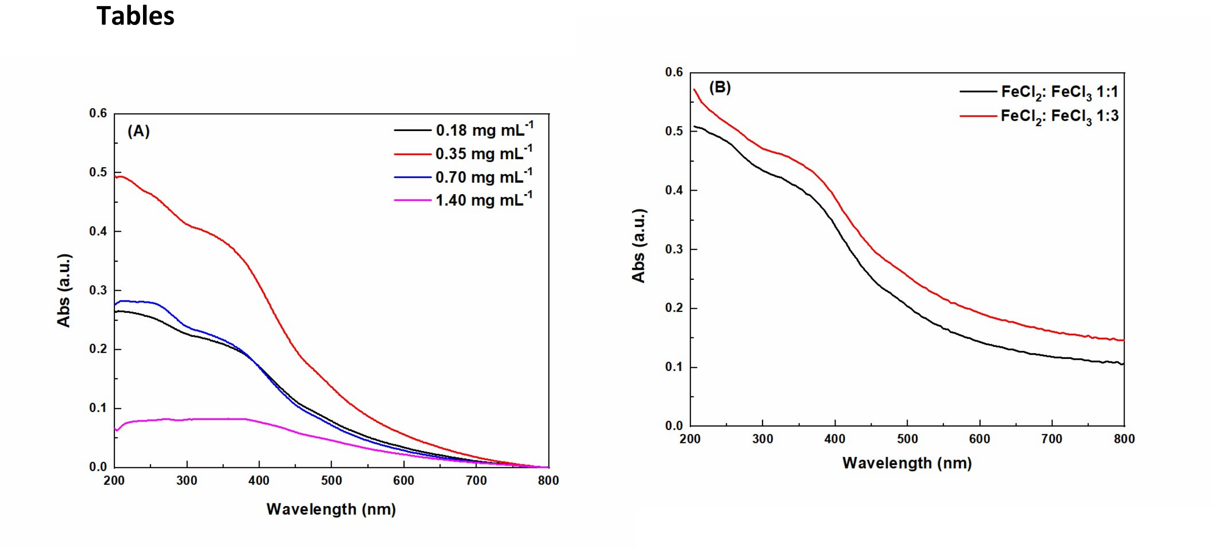

Figure S1. UV-Vis spectra of MbG for (A) different initial concentrations of OLE and (B) different initial ratios of $FeCl_2$: $FeCl_3$.

The ideal concentration of OLE attached to bG for the formation of MbG nanocomposite was 0.35 mg mL^{-1} as determined by the UV-Vis spectra (Figure S1A). At this concentration, the spectrum exhibited a pronounced peak at 380 nm, indicative of successful iron nanoparticle formation. This suggests effective stabilization and reduction facilitated by the phenolic compounds and reducing sugars in OLE [4]. In contrast, lower or higher concentrations resulted in less distinct peaks, likely due to insufficient interaction between bG and the iron precursors or excessive aggregation of nanoparticles, which can obscure spectral features.

Different salt ratios were also tested for the synthesis of MbG. The UV-Vis spectra (Figure S1B) demonstrated distinct absorption peaks corresponding to the formation of iron nanoparticles, with the 1:3 salt ratio exhibiting the most pronounced peak intensity. This indicates optimal conditions for nanoparticle synthesis, as the balanced availability of both iron precursors revealed a defined peak at 380 nm.

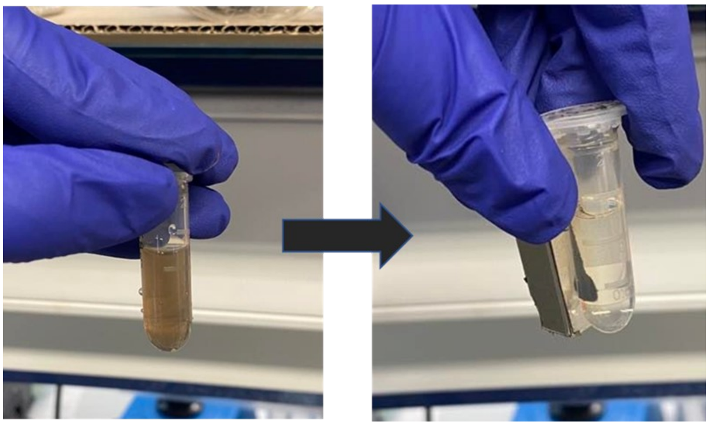

Figure S2. Images before and after magnetization of MbG.

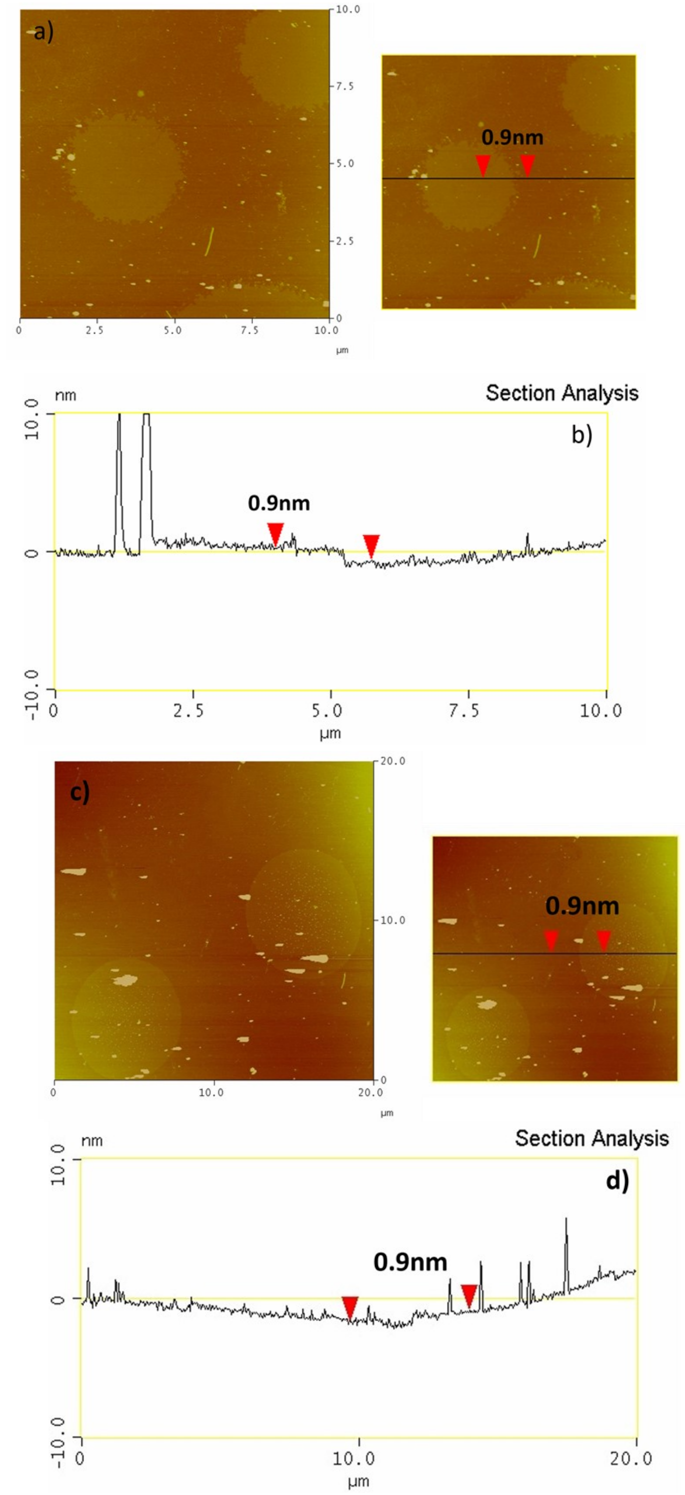

Figure S3. a) AFM images of bG-OLE, b) the height profile of bG-OLE nanosheets, c) AFM images of MbG, and d) the height profile of MbG nanosheets.

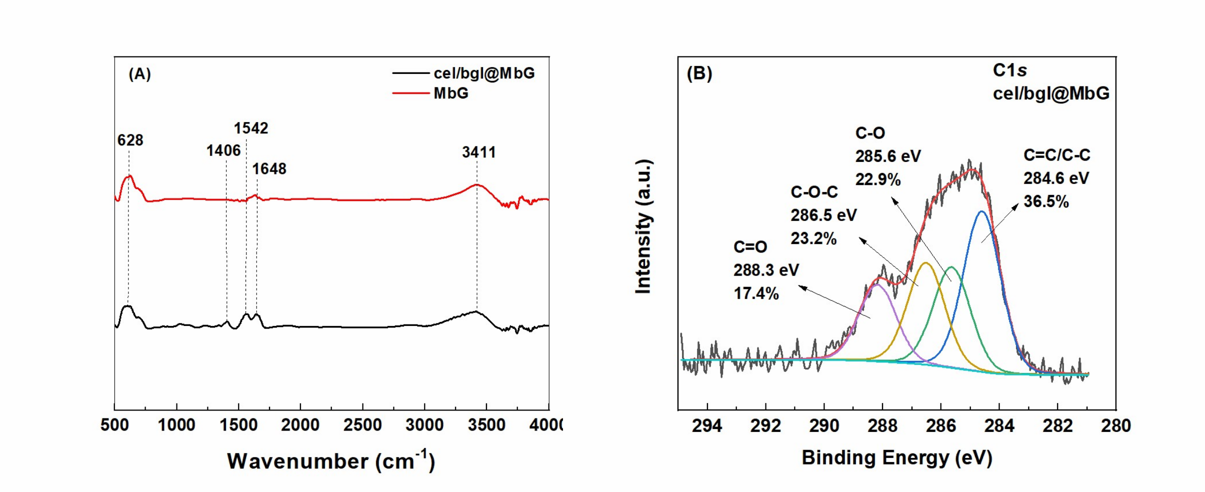

Figure S4. (A) FTIR spectra of MbG and bgl/cel@MbG, (B) C1*s* photoelectron peak of cel/bgl@MbG.

Table S1. Effect of enzyme-to-nanosupport mass ratio on immobilized cel. All analyses were conducted in triplicate, and the results are shown with the associated standard deviations (SD).

mg MbG: mg cel	Immobilization yield (%)	SD	Specific Activity (U mg $^{-1}$)	SD
1:1	7.89	1.70	0.020	0.015
1:2	11.11	2.12	0.050	0.010
1:4	27.42	0.63	0.187	0.025
1:8	28.18	1.80	0.230	0.030

Table S2. Effect of enzyme-to-nanosupport mass ratio on immobilized bgl. All analyses were conducted in triplicate, and the results are shown with the associated standard deviations (SD).

mg MbG: mg bgl	Immobilization yield (%)	SD	Specific Activity (U mg $^{-1}$)	SD
1:0.021	99.78	0.10	0.070	0.015
1:0.032	99.94	0.10	0.150	0.010
1:0.040	99.00	1.00	0.149	0.010
1:0.050	98.88	1.00	0.148	0.005

Table S3. Effect of immobilization incubation time on cel@MbG. All analyses were conducted in triplicate, and the results are shown with the associated standard deviations (SD).

Immobilization time of cel (h)	Immobilization yield (%)	SD	Specific Activity (U mg $^{-1}$)	SD
1	30.52	2.80	0.147	0.010
2	62.26	0.79	0.218	0.001
4	76.12	0.68	0.303	0.001
6	76.00	1.00	0.227	0.010

Table S4. Effect of immobilization incubation time on bgl@MbG. All analyses were conducted in triplicate, and the results are shown with the associated standard deviations (SD).

Immobilization time of bgl (h)	Immobilization yield (%)	SD	Specific Activity (U mg^{-1})	SD
1	99.94	0.79	0.150	0.001
2	99.13	0.68	0.120	0.005
4	98.82	0.11	0.080	0.001
6	98.85	0.3	0.080	0.002

Table S5. Effect of simultaneous and sequential immobilization on the activity of the co-immobilized nanobiocatalyst, the immobilization yield of bgl, and the immobilization yield of cel. All analyses were conducted in triplicate, and the results are shown with the associated standard deviations (SD).

Immobilization form	Immobilization yield (%) of bgl	SD	Immobilization yield (%) of cel	SD	Specific activity (units mg^{-1})	SD
cel priority	99.18	0.68	65.72	0.93	0.204	0.002
bgl priority	99.79	0.60	64.81	0.60	0.284	0.002
Simultaneous immobilization (4h)	59.67	2.40	67.1	1.27	0.287	0.005
Simultaneous immobilization (1h)	63.76	0.78	27.21	2.70	0.328	0.005

Table S6. Comparison of cellulase immobilization systems showing various immobilization carriers and methods.

Immobilization carrier (enzyme)	Method of Immobilization	Substrate	Activity (U $mg_{nanobiocatalyst}^{-1}$)	Reference
Mesoporous biochar (cel)	Physical adsorption	CMC	0.033	5
Carbon nanotubes and sodium alginate (cel)	Entrapment	CMC	0.350	6
Magnetic graphene oxide (cel)	Covalent	Filter paper	0.044	7
Magnetic particles (cel)	Covalent	Filter paper	0.015	8
Magnetic particles (cel)	Covalent	CMC	0.350	9
MbG (cel)	Physical adsorption	CMC	0.150	This work
MbG (cel + bgl)	Physical adsorption	CMC	0.330	This work

Table S7. Effect of pH of free cel, cel@MbG, and cel/bgl@MbG. All analyses were conducted in triplicate, and the results are shown with the associated standard deviations (SD).

pH	Residual Activity of cel (%)	SD	Residual Activity of cel@MbG (%)	SD	Residual Activity of bgl (%)	SD	Residual Activity of bgl @MbG(%)	SD	Residual Activity of cel/bgl @MbG(%)	SD
4.0	81.73	0.33	73.53	1.72	15.13	0.76	88.40	4.11	66.71	1.72
4.5	100.00	1.50	94.07	4.10	26.93	0.77	82.50	5.23	68.29	4.10
5.0	92.60	0.99	100.00	0.33	86.46	5.60	82.10	0.10	100.00	0.33
5.5	75.82	0.33	78.66	4.08	100.00	0.10	100.00	3.90	67.23	4.08

Table S8. Effect of temperature of free cel, cel@MbG, and cel/bgl@MbG. All analyses were conducted in triplicate, and the results are shown with the associated standard deviations (SD).

Temperature (°C)	Residual Activity of cel (%)	SD	Residual Activity of cel@MbG (%)	SD	Residual Activity of bgl (%)	SD	Residual Activity of bgl @MbG(%)	SD	Residual Activity of cel/bgl @MbG(%)	SD
40	42.24	0.44	38.43	0.77	25.88	0.26	49.15	0.20	26.49	0.70
50	73.27	2.13	82.97	3.45	33.51	0.32	68.08	2.95	43.48	0.50
60	100.00	2.45	100.00	4.99	63.89	6.80	77.12	0.10	100.00	0.76
70	76.40	1.86	78.66	1.53	100.00	2.80	100.00	0.10	61.28	0.60

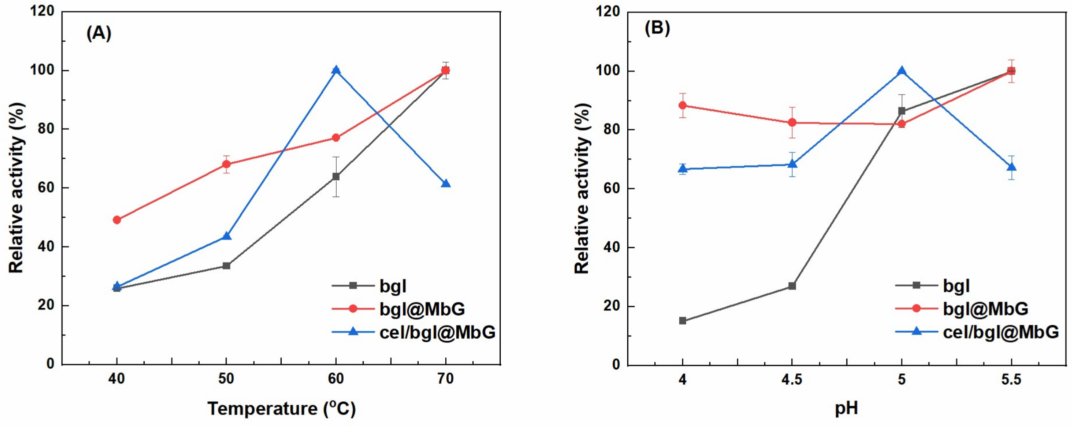

Figure S5. Effect of (A) temperature and (B) pH on the activity of free bgl, bgl@MbG and cel/bgl@MbG. One hundred percent indicates the highest activity exhibited by either the free enzyme or the nanobiocatalyst tested each time. All analyses were conducted in triplicate, and the results are shown with the associated standard deviations. (In some cases, the standard deviation was <1% in relative activity, and thus error bars are not possible to be seen).

Table S9. Reusability of the immobilized nanobiocatalytic systems for the hydrolysis of CMC. All analyses were conducted in triplicate, and the results are shown with the associated standard deviations (SD).

Number of cycles	Remaining activity (%) of cel@MbG	SD	Remaining activity (%) of cel/bgl@MbG	SD
1st	100.00	0.72	100.00	2.31
2nd	55.22	0.17	79.23	1.30
3rd	31.70	0.16	39.62	1.50
4th	23.52	0.15	32.47	2.10

Table S10. Reusability of the immobilized nanobiocatalytic systems for the hydrolysis of Avicel. All analyses were conducted in triplicate, and the results are shown with the associated standard deviations (SD).

Number of cycles	Remaining activity (%) of cel@MbG	SD	Remaining activity (%) of cel/bgl@MbG	SD
1st	100.00	1.50	100.00	2.00
2nd	27.86	2.00	42.31	1.50
3rd	2.31	0.50	11.54	1.70
4th	0.00	0.00	7.69	1.30

Table S11. Effect of the flow rate on the productivity of cel@MbG microreactor. All analyses were conducted in triplicate, and the results are shown with the associated standard deviations (SD).

Flow rate (μL min^{-1})	Productivity $\mu g_{(glucose)} min^{-1} \mu g_{(cel@MbG)}^{-1}$	SD
1	0.042	0.006
2	0.067	0.001
4	0.038	0.002
6	0.001	0.000

References

1 A. V. Chatzikonstantinou, A. Giannakopoulou, S. Spyrou, Y. V. Simos, V. G. Kontogianni, D. Peschos, P. Katapodis, A. C. Polydera and H. Stamatis, *Environ. Sci. Pollut. Res.*, 2022, **29**, 29624–29637.

2 M. Bradford, *Anal. Biochem.*, 1976, **72**, 248–254.

3 G. L. Miller, *Anal. Chem.*, 1959, **31**, 426–428.

4 M. Jamzad and M. Kamari Bidkorpeh, *J. Nanostructure Chem.*, 2020, **10**, 193–201.

5 C. hui Zhu, Z. Fang, T. chao Su, X. kang Li and Q. ying Liu, *Cellulose*, 2018, **25**, 2473–2485.

6 L. J. Li, W. J. Xia, G. P. Ma, Y. L. Chen and Y. Y. Ma, *AMB Express*, DOI:10.1186/s13568-019-0835-0.

7 F. R. Paz-Cedeno, J. M. Carceller, S. Iborra, R. K. Donato, A. P. Godoy, A. Veloso de Paula, R. Monti, A. Corma and F. Masarin, *Renew. Energy*, 2021, **164**, 491–501.

8 J. Alftrén and T. J. Hobley, *Biomass and Bioenergy*, 2014, **65**, 72–78.

9 J. Pei, Y. Huang, Y. Yang, H. Yuan, X. Liu and C. Ni, *J. Inorg. Organomet. Polym. Mater.*, 2018, **28**, 1624–1635.
